# Advances of Mussel-Inspired Nanocomposite Hydrogels in Biomedical Applications

**DOI:** 10.3390/biomimetics8010128

**Published:** 2023-03-22

**Authors:** Haohua Ma, Xin Qiao, Lu Han

**Affiliations:** Laboratory for Marine Drugs and Bioproducts, School of Medicine and Pharmaceutics, Ocean University of China, Qingdao 266005, China

**Keywords:** mussel-inspired hydrogel, nanocomposite hydrogel, nanomaterials, biomedical applications

## Abstract

Hydrogels, with 3D hydrophilic polymer networks and excellent biocompatibilities, have emerged as promising biomaterial candidates to mimic the structure and properties of biological tissues. The incorporation of nanomaterials into a hydrogel matrix can tailor the functions of the nanocomposite hydrogels to meet the requirements for different biomedical applications. However, most nanomaterials show poor dispersion in water, which limits their integration into the hydrophilic hydrogel network. Mussel-inspired chemistry provides a mild and biocompatible approach in material surface engineering due to the high reactivity and universal adhesive property of catechol groups. In order to attract more attention to mussel-inspired nanocomposite hydrogels, and to promote the research work on mussel-inspired nanocomposite hydrogels, we have reviewed the recent advances in the preparation of mussel-inspired nanocomposite hydrogels using a variety of nanomaterials with different forms (nanoparticles, nanorods, nanofibers, nanosheets). We give an overview of each nanomaterial modified or hybridized by catechol or polyphenol groups based on mussel-inspired chemistry, and the performances of the nanocomposite hydrogel after the nanomaterial’s incorporation. We also highlight the use of each nanocomposite hydrogel for various biomedical applications, including drug delivery, bioelectronics, wearable/implantable biosensors, tumor therapy, and tissue repair. Finally, the challenges and future research direction in designing mussel-inspired nanocomposite hydrogels are discussed.

## 1. Introduction

Hydrogels are three-dimensional (3D) networks of crosslinked hydrophilic polymers, which are similar to soft tissues owing to their porous structure, high water content, and controllable properties. Therefore, hydrogels have a great potential in biomedical applications [1,2,3,4,5,6,7,8,9]. However, hydrogels usually have weak mechanical properties and cannot meet the requirements for different biomedical applications. Incorporation of functional nanomaterials into hydrogels can introduce various new properties to the hydrogels [10,11]. In addition, hydrogels can also effectively improve the retention effect of nanomaterials in vivo [12,13]. Nanomaterials can be classified into three main types based on their dimensionality (size and morphology): zero-dimensional (0D), one-dimensional (1D), and two-dimensional (2D) [14,15,16]. The 0D nanomaterials are solid, porous, and hollow structures, such as mesoporous silica nanoparticles (NPs) [17], metal-organic frameworks [18], hydroxyapatite NPs [19], iron oxide magnetic NPs [20], silver NPs [21], and conductive polymer-based NPs [22]. The 1D nanomaterials are nanowires, nanorods, or nanotubes, with a large length-to-diameter ratio, such as carbon nanotubes (CNTs) [23], gold nanorods (Au NRs) [24], and cellulose nanofibers (CNFs) [25]. The 2D nanomaterials are nanosheets with monolayer or multilayer structures and relatively large diameter-to-thickness ratios, such as graphene oxide (GO) [26], clay [27], talc, phosphate-based nanosheets, and metal carbides and nitrides [28]. However, it is generally difficult to achieve a uniform distribution of nanomaterials in hydrogels due to their weak interactions with the polymer chains.

In nature, mussels can form a strong adhesion to various substrates in wet or underwater conditions, which relies on their secreted adhesive proteins containing a catecholic amino acid (3,4-dihydroxy-L-phenylalanine, DOPA) [29]. This unique adhesive performance of mussels simulates the mussel-inspired chemistry, which provides a mild and biocompatible approach in material surface engineering due to the high reactivity and universal adhesive property of the catechol group [30]. The catechol group consists of two adjacent hydroxyl groups that are attached to an aromatic ring [31], which enables the formation of catechol-mediated interactions, such as to π–π interactions, cation-π, hydrogen bond, DOPA-quinone coupling interaction, catechol-metal coordination, catechol -borate complexation, and Michael addition or Schiff-base reactions [32,33,34,35,36,37]. Since Messersmith et al. [38,39] reported mussel-inspired multifunctional polydopamine (PDA) coatings, materials containing catechol groups have been widely developed. The catechol-derivative groups possess high activities and binding affinities that can control the interfacial chemistry of nanoparticles to improve their dispersion in aqueous conditions [40]. Mussel-inspired hydrogels have also been widely studied for applications in regenerative medicine and tissue engineering [41,42]. For example, catechol groups can endow hydrogels with excellent adhesive properties, enabling the hydrogel to bind closely to the surrounding tissues after implantation without the need for surgical adhesives [43]. Furthermore, catechol groups can promote interactions between hydrogels and cells owing to their good cell affinity, which is conducive to the growth of biological tissues [44,45]. Moreover, catechol groups can scavenge free radicals, endowing hydrogels with an anti-oxidative ability [46,47]. Thus, the combination of catechol groups and functional nanomaterials is crucial for developing multifunctional hydrogels.

**Scheme 1:**
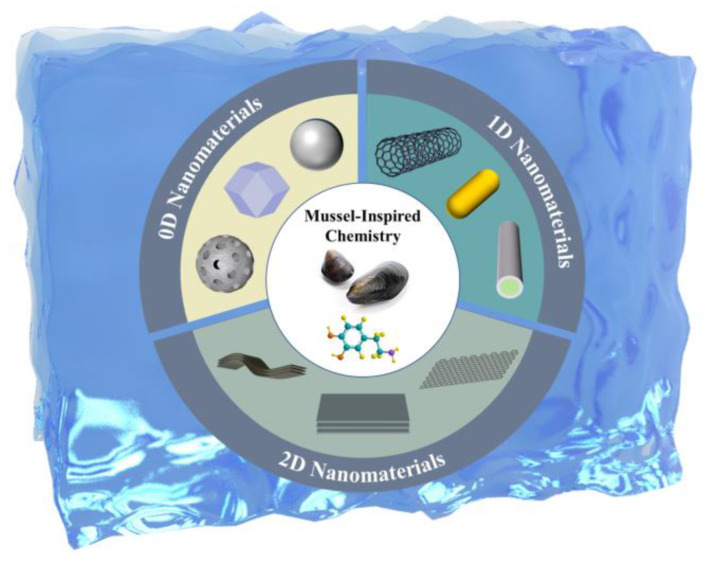
Overview of the mussel-inspired nanocomposite hydrogels composed of nanomaterials with different dimensions.

This review summarizes the research progress on mussel-inspired nanocomposite hydrogels based on 0, 1, and 2D nanomaterials, and their biomedical applications, including drug delivery, bioelectronics, wearable/implantable biosensors, tumor therapy, and tissue repairing biomaterials (Scheme 1). We focused on the major design parameters of each nanocomposite hydrogel with an emphasis on the principle behind the selection of the nanofiller, its function when added to the matrix, the gelation mechanism, and the physicochemical, mechanical, and biological properties of the resulting nanocomposite hydrogels (Table 1). Furthermore, we discussed the current challenges of mussel-inspired nanocomposite hydrogels and provided prospects for future research.

## 2. Mussel-Inspired 0D Nanomaterials-Loaded Hydrogels

Zero-dimensional (0D) nanomaterials have an almost identical size at the nanometer level (i.e., <100 nm) in all three dimensions. Typical 0D nanomaterials are inorganic nanoparticles, magnetic nanoparticles, metal nanoparticles, and polymer-based nanoparticles.

### 2.1. Hydroxyapatite Nanoparticles (HA NPs)

**Figure 1:**
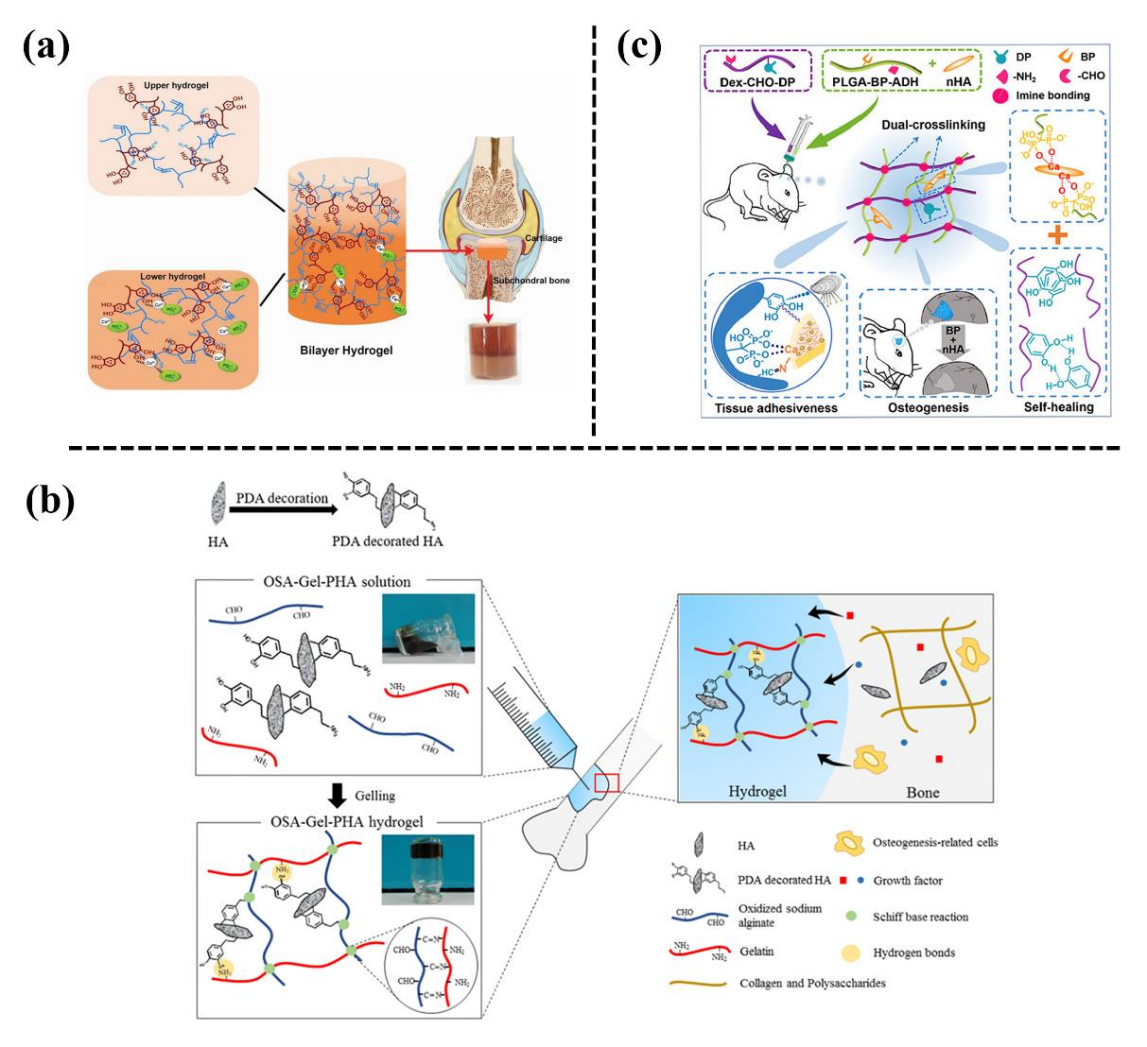
(**a**) Illustrative diagram for the preparation of a mussel-inspired GelMA hydrogel with in situ mineralization of nano-hydroxyapatite to repair osteochondral defects [48]. (**b**) Incorporation of PDA-modified hydroxyapatite nanoparticles (PHA NPs) into an oxidized sodium alginate/gelatin hybrid hydrogel for bone regeneration [49]. (**c**) Preparation of mussel-inspired bisphosphonated injectable nanocomposite hydrogels with adhesive, self-healing, and osteogenic properties [50].

Hydroxyapatite (HA) is a natural bioceramic material that is an inorganic mineral component of bone tissue. Nanometre HA refers to the nanometre-sized (1–100 nm) HA NPs. HA NPs can be used as nanoreinforcing agents to enhance osteoconductivity of the polymer hydrogel. In order to achieve a uniform distribution of HA NPs inside the polymer network, a mussel-inspired strategy has been widely used to modify the HA NPs prior to being incorporated into the hydrogel. For instance, Gan et al. [48] developed a bilayer hydrogel to repair osteochondral defects (Figure 1a). The upper layer consisted of a GelMA-PDA hydrogel, which served as the cartilage repair layer, and the lower layer consisted of a GelMA-PDA/calcium phosphate (GelMA-PDA/HA) hydrogel, which served as the subchondral bone repair layer. Consequently, the bilayer hydrogel simultaneously promoted bone and cartilage tissue regeneration after being implanted into a full-layer cartilage defect of a rabbit knee joint. Liu et al. [49] also prepared an injectable hydrogel for bone repair by introducing PDA-modified nHA NPs (PHA) into a sodium alginate (OSA)/gelatin (Gel) hybrid network (Figure 1b). The addition of PHA increased the ultimate compressive strength of the hydrogel. In addition, the OSA-Gel-PHA hydrogel significantly promoted the adhesion, proliferation, and differentiation of bone marrow mesenchymal stem cells in vitro, and also repaired bone tissue in a rabbit bone defect model. In another study, Wang et al. [50] designed an injectable mussel-inspired nanocomposite hydrogel based on HA NPs, bisphosphonated poly(L-glutamic acid), and aldehyde-catechol bis-functionalized dextronic anhydride for bone tissue engineering (Figure 1c). The catechol groups, bisphosphate ligands (BPs), and aldehyde groups endowed the hydrogels with excellent tissue adhesion, while the BP and nHA effectively promoted proliferation, migration, and osteogenesis differentiation. The results of a rat cranial defect proved the bone regeneration ability of the injectable hydrogels. Based on these studies, it can be said that mussel-inspired addition of HA NPs not only facilitates the distribution of HA NPs, to enhance the mechanical properties of the hydrogel, but also improves cell/tissue affinity to stimulate osteogenesis for the nanocomposite hydrogel. Therefore, the HA NPs-loaded hydrogels could be used for hard tissue engineering for bone grafts.

### 2.2. Iron Oxide Magnetic Nanoparticles (MNPs)

**Figure 2:**
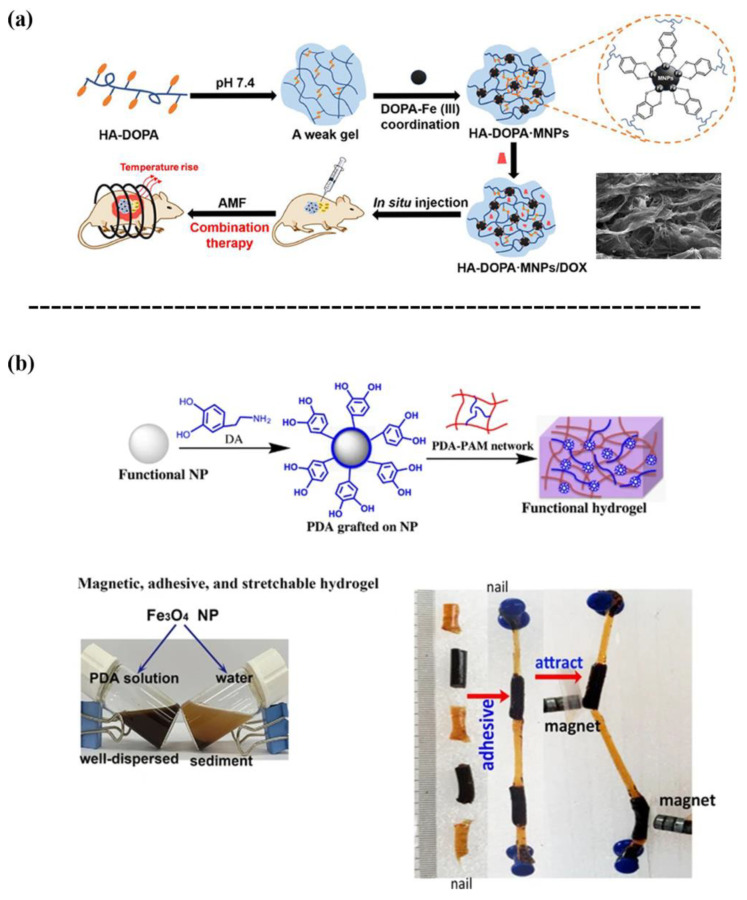
(**a**) Schematic of the preparation of magnetic nanocomposite hydrogels (HA-DOPA MNPs) [51]. (**b**) Magnetic hydrogel formed by incorporating PDA-grafted-Fe_3_O_4_ NPs into a PAM hydrogel [52].

Iron oxide MNPs are classified as magnetic materials, and are generally embedded within a polymer network to achieve magnetic-based nanocomposite hydrogels that can remotely respond to external magnetic fields and can be used for biosensing, diagnostic, and actuators applications [96,97,98,99]. However, iron oxide MNPs have limited interactions with polymer chains, which requires surface modification prior to fabricating the hydrogel. The surface chemistry based on the coordination of catechol-Fe(III) is one crucial strategy for the synthesis of magnetic-based nanocomposite hydrogels. For instance, Dai et al. [51] reported a catechol-Fe(III) coordination hydrogel composed of dopamine-conjugated hyaluronic acid (HA-DOPA) and iron oxide MNPs (Figure 2a). The MNPs in the hydrogel not only served as a structural crosslinking agent to enhance the hydrogel, but also a magnetothermal conversion agent for on demand release of doxorubicin (DOX). Consequently, the HA-DOPA-MNPs/DOX hydrogel exhibited anticancer effects through the combined release of DOX and induction of hyperthermia in the tumor cells. In our previous study [52], we designed a magnetic hydrogel with a high flexibility, self-healing ability, and tissue adhesiveness by incorporating PDA-grafted-Fe_3_O_4_ NPs into a PAM hydrogel (Figure 2b). In short, the iron oxide MNPs-loaded hydrogels will generate magnetic responses and heat for drug release when they are exposed to an external magnetic field.

### 2.3. Mesoporous Silica Nanoparticles

**Figure 3:**
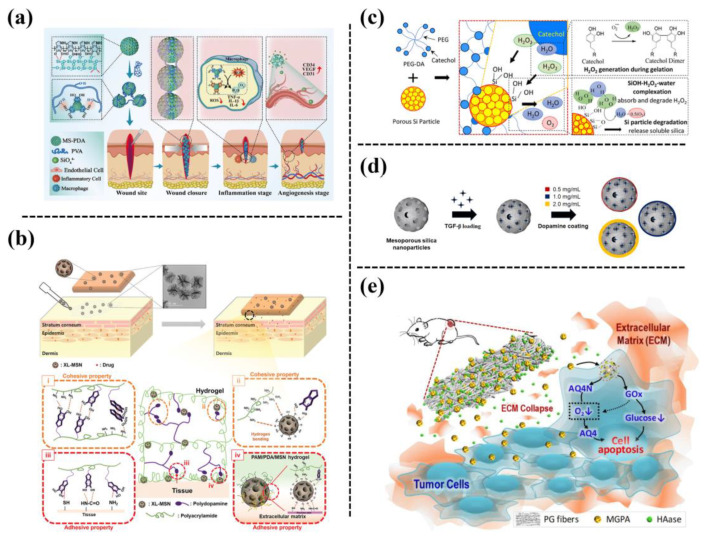
(**a**) Schematic of preparing a PVA entangled porous nanoadhesive system of PDA/silica with an ROS scavenging/angiogenesis ability for efficient skin wound closure [53]. (**b**) Schematic of adhesive hydrogel patch consisting of PAM/PDA hydrogels embedded with extra-large pore mesoporous silica nanoparticles (XL-MSNs) [54]. (**c**) Fabrication of a catechol-containing polyethylene glycol-based adhesive (PEG-DA) formulated with silica NPs to control the released amount of H_2_O_2_ [55]. (**d**) Fabrication of PDA-coated mesoporous silica NPs that can be stably incorporated within macroporous μRB scaffolds to allow the tunable release of transforming growth factor (TGF-β3) [56]. (**e**) Schematic illustration of combination therapy based on hierarchical PGH@MGPA composite fibers [57].

Mesoporous silica nanoparticles (silica NPs) are widely used as drug delivery systems owing to their unique properties, such as high specific surface area, large pore volume, controllable morphology, and particle size [100,101,102]. Thus, various nanocomposite hydrogels containing silica NPs have been developed for adhesive and drug delivery. For example, Huang et al. [53] developed an adhesive comprised of polyvinyl alcohol (PVA) and PDA-hybrid mesoporous silica NPs (MS-PDA-NPs), which was formed based on the hydrogen bonding between the functional groups on MS-PDA-NPs and abundant hydroxyl groups on the PVA chain (Figure 3a). Thus, such a mesoporous silica NP-reinforced PVA hydrogel can be used for wound closure. A biocompatible bioadhesive was also fabricated by embedding extra-large pore mesoporous silica NPs into polyacrylamide/polydopamine (PAM/PDA) hydrogels [54] (Figure 3b). The incorporation of silica NPs enhanced the mechanical strength and tissue adhesiveness to skin due to molecular interactions between silica NPs and polymer chains. It was proved that a silica NPs-PAM/PDA hydrogel could be used as an adhesive patch for transdermal drug delivery. Another tissue adhesive was reported that used porous silica NPs to reinforce catechol-functionalized polyethylene glycol [55] (Figure 3c).

Mesoporous silica NPs are also promising drug delivery carriers. The drugs can be loaded into the pores of the silica NPs through diffusion with minimal interference with the biological activity of the drugs. For example, Barati et al. [56] employed PDA-coated mesoporous silica NPs for the encapsulation of transforming growth factor (TGF-β3), and then incorporated the TGF β3-loaded silica NPs into gelatin scaffolds to accelerate cartilage regeneration (Figure 3d). Their results showed that the PDA coating on the surface of the mesoporous silica NPs prevented the burst release of TGF-β3, and the sustained release of TGF-β3 could enhance MSC-based cartilage formation in vivo. The PDA-coated mesoporous silica NPs were also used for dual drug encapsulation, in which glucose oxidase was loaded inside the NPs while anoxic-activated prodrug (AQ4N) was adsorbed onto the surface [57]. Then the drug-loaded NPs were incorporated into polycaprolactone/gelatin to prepare a biodegradable fiber scaffold (PGH@MGPA) as an implantable drug delivery system for synergistic cancer therapy (Figure 3e). The porous silica NPs have a high specific surface area and strong interactions with catechol groups, thus improving the curing rate, mechanical properties, and bonding strength of the adhesive. In addition, the porous silica NPs degraded into soluble Si, which promoted cell proliferation.

### 2.4. Metal-Organic-Framework

**Figure 4:**
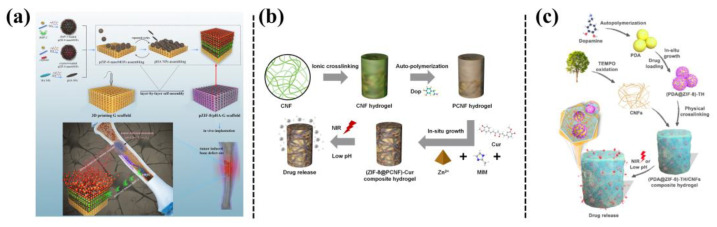
(**a**) Schematics of assembling polydopamine (PDA)-hybridized ZIF-8 (pZIF-8 nanoMOFs) with PDA-modified hydroxyapatite NPs (pHA NPs) on a 3D printed gelatin scaffold, with dual functions of anticancer and bone formation capabilities [58]. (**b**) Preparation of a ZIF-8@PCNF composite hydrogel for on-demand drug release by responding to pH and NIR light irradiation [59]. (**c**) Fabrication of a PDA@ZIF-8/CNFs composite hydrogel and its drug delivery behavior upon NIR light irradiation or under pH variation [60].

Nanocarriers based on MOFs have received significant interest owing to their large surface area, high porosity, and possibility of designing organic ligands for various applications. Zeolite imidazole MOF (ZIF-8), synthesized using zinc ions and 2-methylimidazole, is one of the most widely used and promising MOFs, which can be used as a biocompatible, degradable, and flexible drug carrier. For example, Han et al. [58] reported producing PDA-hybridized nanosized ZIF-8 (pZIF-8 nanoMOFs), through catechol-controlled chemistry, which possessed versatile adhesiveness, a porous structure, high stability under physiological conditions, and pH-/oxidative dual-responsiveness. Thus, pZIF-8 nanoMOFs were highly efficient for encapsulation of both bone morphogenetic protein-2 (BMP-2) and cisplatin. Then the drug-loaded pZIF-8 nano-MOFs were assembled with PDA-modified HA NPs on a 3D-printed gelatin hydrogel scaffold, which achieved on demand release of the cisplatin, responding to the local tumor microenvironment to inhibit tumor growth, which enabled the sustained release of BMP-2, to maintain effective long-term osteogenic effects (Figure 4a). Liu et al. [59] loaded curcumin into ZIF-8 MOFs and obtained nanocomposite hydrogels with pH responsiveness and NIR-photosensitive drug release ability, by incorporating curcumin-ZIF-8 MOFs into PDA-modified cellulose nanofiber hydrogels (Figure 4b). By being co-incubated with normal buffalo rat liver cells (BRL) and liver cancer cells (HepG2), the hydrogel had anticancer activity against HepG2 cells, but was not toxic to normal BRL cells. In order to improve the applicability of ZIF-8 in the powder crystallization state, and to increase the drug loading of PDA NPs, Liu et al. obtained PDA@ZIF-8 by in situ growth of ZIF-8 on the surface of PDA NPs, and then mixing PDA@ZIF-8 into a cellulose nanofibril (CNFs)-based hydrogel (Figure 4c). The PDA@ZIF-8 exhibited a photothermal effect from the PDA NPs, and pH-responsiveness from ZIF-8, and therefore, the resulting PDA@ZIF-8/CNFs nanocomposite hydrogel achieved pH/NIR radiation-dependent release of tetracycline hydrochloride [60].

### 2.5. Silver Nanoparticles (Ag NPs)

**Figure 5:**
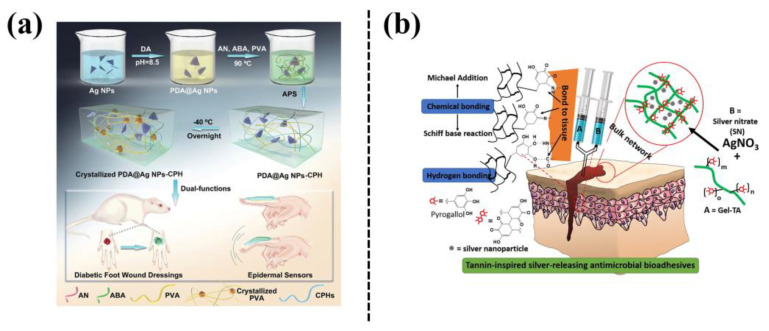
(**a**) Synthesis of a PDA@Ag NPs-CPH hydrogel and its applications as an epidermal sensor and diabetic-foot-wound dressing [61]. (**b**) Tannin-inspired antimicrobial bioadhesives crosslinked by silver nitrate (SN) [62].

Silver nanoparticles (Ag NPs) exhibit a high thermal stability and a broad spectrum of antibacterial activities, which has attracted much attention in the preparation of nanocomposite hydrogels [103]. However, the aggregation of Ag NPs results in their uneven dispersion in a hydrogel network. Mussel-inspired PDA, with catechol and amine groups, is considered as a reducing or stabilizing agent for preparing Ag NPs in a simple and environmentally friendly manner, and the as-prepared Ag NPs serve as nanofillers to endow the hydrogels with multiple functions. For example, Zhao et al. [61] prepared a hydrogel (PDA@Ag NPs-CPHs) by incorporating polydopamine-modified Ag NPs into polyaniline-PVA hydrogel (Figure 5a). The PDA@Ag NPs-CPHs hydrogel exhibited adhesiveness, conductivity, and antibacterial properties, and can be used in wearable and implantable biomedical devices. The PDA@Ag NPs-CPHs retained the antibacterial and electroactive properties and demonstrated significant therapeutic effects on diabetic-foot wounds by promoting angiogenesis, accelerating collagen deposition, and inhibiting wound infection. The Ag NPs can also be formed in situ in catechol-groups-modified hydrogels. For example, a gelatin-tannic acid (Gel-TA) hydrogel was formed through a Michael addition reaction, and the silver nitrate was reduced in situ by TA to Ag NPs as crosslinking agents [62] (Figure 5b). The hydrogel thus exhibited excellent wet tissue adhesion and cytocompatibility, as well as antibacterial and antifungal properties.

**Figure 6:**
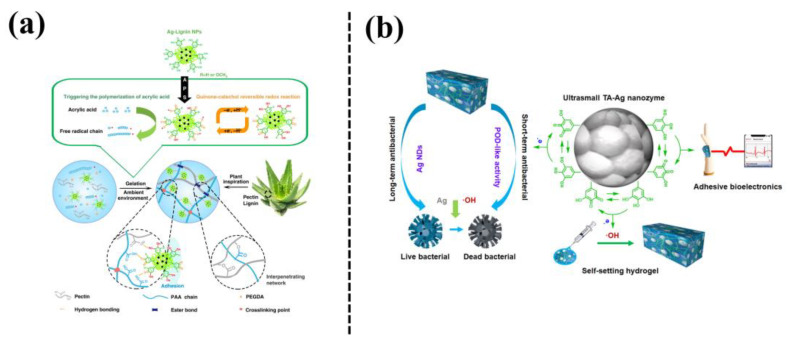
(**a**) Fabrication process of plant-inspired catechol-chemistry-based hydrogels by incorporation of the silver-lignin NPs to achieve self-adhesive, tough, and antibacterial properties [63]. (**b**) The TA-Ag nanozyme-loaded polyacrylic acid hydrogel (TA-Ag-PAA) with conductive and self-setting properties [64].

In addition, phenol/catechol groups-chelated Ag NPs can form a dynamic redox system, which can not only trigger hydrogel gelation but also endow the hydrogel with long-term tissue adhesiveness. For example, a plant-inspired adhesive hydrogel was fabricated based on a dynamic catechol redox system created by catechol-containing lignin-chelated Ag (Ag-Lignin) NPs [63] (Figure 6a). The Ag-Lignin NPs catalyzed ammonium persulfate to generate free radicals and initiated the polymerization of the hydrogel. The hydrogel showed long-term repeatable adhesion because Ag-lignin NPs maintained a dynamic balance of the catechol groups. In another study, Jia et al. [64] reported mussel-inspired TA-Ag nanozymes by in situ reduction of ultrasmall Ag NPs with tannic acid (TA) (Figure 6b). The TA-Ag nanozymes exhibited peroxidase activity, which triggered self-setting of the hydrogel without external stimuli. The as-prepared hydrogel could be used as an adhesive and antibacterial bioelectrode to detect bio-signals, and also as a wound dressing to promote the regeneration of skin tissue, with a healing rate of up to 90% in a rat model of full-thickness skin defect. In mussel-inspired nanocomposite hydrogels based on Ag NPs, the catechol-containing agents not only serve as reducing agents to obtain Ag NPs for achieving antibacterial ability, but also can be used to create a dynamic redox system inside the hydrogel for maintaining long-term adhesion.

### 2.6. Polydopamine Nanoparticles (PDA-NPs)

**Figure 7:**
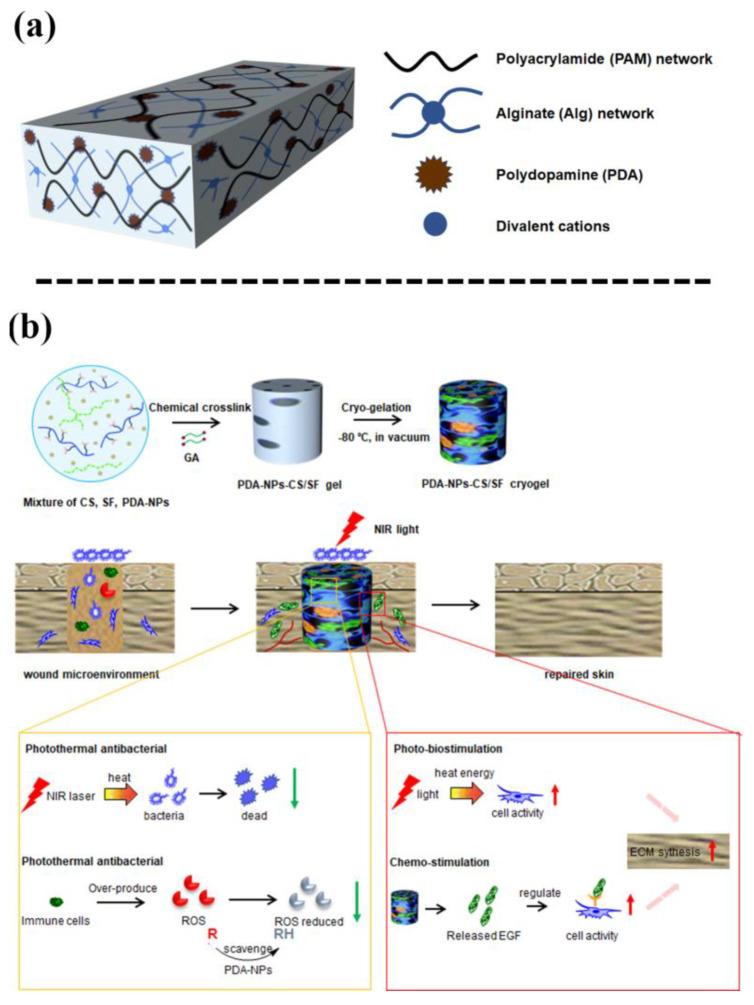
(**a**) Incorporating PDA-NPs into a dual-network of PAM and alginate to form underwater adhesive PAM-Alg-PDA hydrogel [65]. (**b**) Schematics of mussel-inspired PDA-chitosan-silk fibroin cryogel as a photothermal patch for promoting wound regeneration [66].

PDA-NPs, formed by self-polymerization of PDA, have been widely used in diagnosing and treating diseases owing to their excellent properties, such as good adhesiveness, anti-oxidative ability, photothermal conversion ability, drug loading ability, and biocompatibility [104]. PDA-NPs with highly reactive catechol groups can improve the adhesive and mechanical properties of hydrogels. For example, Liang et al. [65] incorporated PDA-NPs into a dual-network of polyacrylamide and alginate, thereby enhancing the adhesive ability of the hydrogel in a seawater environment (Figure 7a). The optimal adhesive strength of the hydrogel in seawater can be as high as 146.84 ± 7.78 kPa. In addition, we also prepared a cryogel for wound dressing by incorporating PDA-NPs into a chitosan (CS) and silk fibroin (SF) hybrid network [66] (Figure 7b). The PDA-NPs endowed the cryogel with high elasticity and flexibility, cell affinity, and strong antibacterial activity under NIR irradiation. Consequently, the PDA-NPs-CS-SF cryogel accelerated wound healing under NIR irradiation.

**Figure 8:**
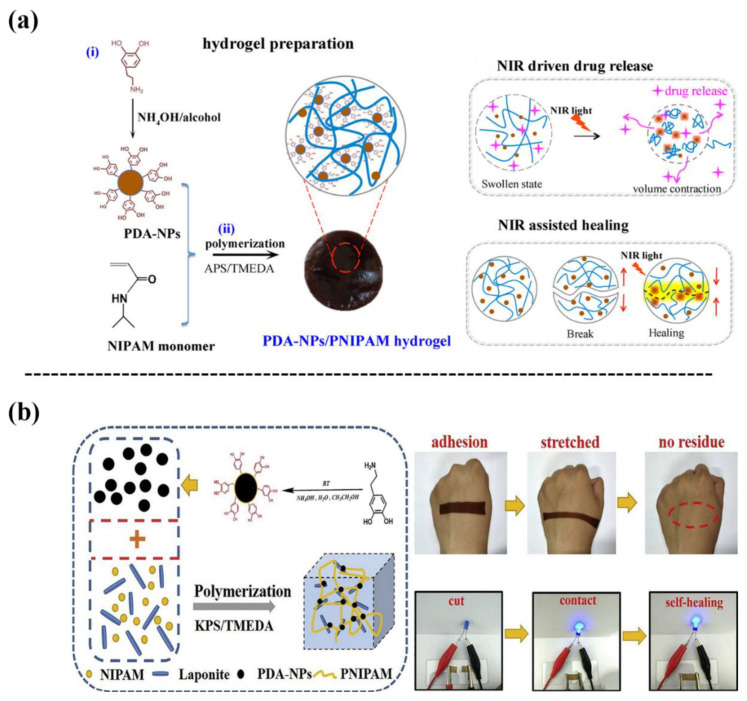
(**a**) Incorporating PDA-NPs into a PNIPAM hydrogel to fabricate a PDA-NPs/PNIPAM hydrogel with NIR responsiveness, self-healing, on-demand drug releasing, and adhesive properties [67]. (**b**) Fabrication of a PDA-NPs and laponite-embedded nanocomposite hydrogel with stretchability, conductivity, dual light- and thermo-responsive, and adhesive properties [68].

Owing to the excellent photothermal conversion ability of PDA-NPs, thermal responsive polymers such as poly(n-isopropyl acrylamide) (PNIPAM) are generally used to develop NIR responsive nanocomposite hydrogels. For a successful example, we designed a NIR responsive hydrogel by introducing PDA-NPs into a PNIPAM/PAM hybrid network [67] (Figure 8a). The photothermal effect of the NIR-responsive PDA-NPs endowed the PNIPAM-based hydrogel with pulsed drug release, NIR drive, and NIR response-self-healing capabilities, in addition to an improved cell affinity and tissue adhesion. Di et al. [68] also synthesized a nanocomposite hydrogel with excellent durability and repeatable adhesion based on PDA-NPs, clay, and PNIPAM (Figure 8b). The hydrogel achieved thermal-responsiveness and local controllable deformation under remote NIR irradiation, owing to the phase transition and volume change of the PNIPAM network.

**Figure 9:**
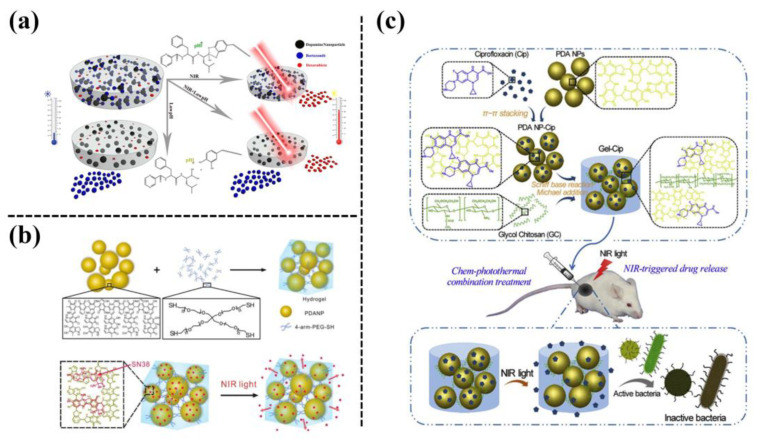
(**a**) Schematic of a multi-stimulus responsive mussel-inspired hybrid hydrogel as a single platform for synergistic anticancer treatment by combining PTT and multidrug chemotherapy [69]. (**b**) Preparation of an NIR-responsive hydrogel by using PDA-NPs as a crosslinking agent to crosslink 4-arm-PEG-SH to realize NIR-triggered drug release [70]. (**c**) Preparation of an NIR light-triggerable thermo-sensitive hydrogel by mixing ciprofloxacin (Cip)-loaded PDA-NPs with glycol chitosan (GC) [71].

In addition, PDA-NPs have a large number of phenolic hydroxyl groups on their surfaces, which can be smart nanocarriers for drug loading by combining their photothermal conversion ability. In a recent report, PDA-NPs were used to load bortezomib (BTZ) and doxorubicin (DOXO), and then the drug-loaded PDA-NPs were incorporated into a PNIPAM-co-PAAM hydrogel (Figure 9a). The PDA-NPs served as photothermal agents, facilitating the controlled release of DOXO to kill the tumor cells under NIR irradiation [69]. Wang et al. [70] also used PDA-NPs as crosslinking agents for mercaptoylated four-arms PEG (4-arms-PEG-SH) to form an injectable PDA/PEG hydrogel for on-demand administration and chemotherapy-photothermal combination therapy (Figure 9b). The anticancer drug 7-ethyl-10-hydroxycamptothecin (SN38) was loaded onto PDA-NPs via π-π interactions and hydrogen bonding. Under NIR irradiation, the PDA/PEG hydrogel achieved the controlled release of SN38 to ablate solid tumors. Using the same method, ciprofloxacin (Cip) was loaded on PDA-NPs and then the Cip-loaded PDA-NPs were mixed with ethylene glycol chitosan to form an injectable hydrogel (Gel-Cip) [71] (Figure 9c). Under NIR irradiation, the Gel-Cip achieved a controlled release of Cip. Simultaneously, NIR irradiation activated the photothermal PDA-NPs, resulting in local high temperatures to damage the bacterial integrity. In a mouse model of skin injury caused by Staphylococcus aureus infection, the Gel-Cip hydrogel promoted the regeneration of blood vessels and hair follicles, along with epidermal thickening and fibroblast proliferation, with the assistance of NIR irradiation. In short, the photothermal conversion ability of PDA-NPs has been widely employed for designing thermal-responsive hydrogel to realize antibacterial, drug responsive release, and tumor killing under NIR irradiation.

### 2.7. Conductive-Polymer Nanoparticles

**Figure 10:**
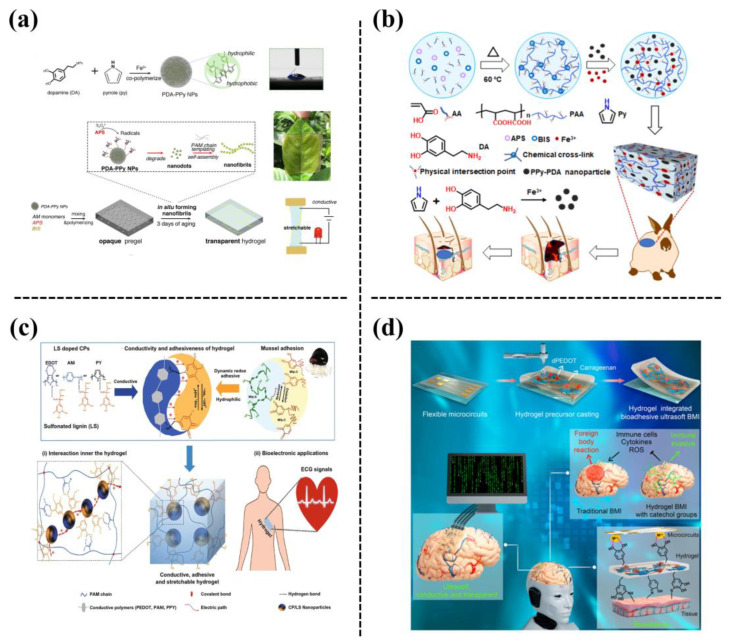
(**a**) A transparent, conductive, stretchable, and adhesive hydrogel was formed by in-situ formation of PDA-PPy nanofibrils in a PAM hydrogel [72]. (**b**) Schematic of the fabrication processes and application of the PPy-PDA/PAA hydrogels [73]. (**c**) Preparation of conductive and adhesive hydrogel based on hydrophilic and redox-active conductive-polymer/sulfonated lignin (CP/LS) NPs [74]. (**d**) A bioadhesive ultra-soft brain-machine interface (BMI) was fabricated based on integration of metallic microcircuits with a dopamine methacrylate-hybridized poly(3,4-ethylenedioxythiophene) nanoparticle (dPEDOT NP)-loaded hydrogel [75].

Conductive polymers (CPs) exhibit unique electronic/ionic conductivity and biocompatibility. These mainly include polypyrrole (PPy), poly(3,4-ethylenedioxythiophene) (PEDOT), polythiophene (PTH), and polyaniline (PANI) [105]. The CPs are designed in the form of nanoparticles that are then incorporated into a polymer network to form conductive hydrogels. However, it is challenging to prepare CP nanoparticle-based hydrogels because the poor water solubility and hydrophobic nature of CPs has limited their integration with hydrophilic hydrogel networks. In our group, we reported a mussel-inspired strategy by using highly hydrophilic and active catechol groups to dope the CPs during processing, which shed light on how to construct hydrophilic CPs nanoparticles and prepare adhesive and conductive hydrogels. For example, a transparent, conductive, stretchable, and self-adhesive hydrogel was designed by the in situ formation of PDA-doped-PPy nanofibrils in a polyacrylamide (PAM) network [72] (Figure 10a). The obtained PDA-PPy-PAM hydrogel can be used in self-adhesive biosensors, to be directly adhered to the human body to detect biosignals about human health. Inspired by the same mechanism, Chen et al. [73] synthesized a PPy-PDA/polyacrylic acid (PAA) hydrogel, which possessed excellent tissue adhesiveness, electrical conductivity, and antioxidant ability (Figure 10b). The hydrogel was used as a wound dressing, as demonstrated in a rabbit wound model. In another study, plant-derived sulfonated lignin was employed for doping a variety of CPs to prepare hydrophilic conductive nanoparticles (CP/LS NPs), and then the CP/LS NPs were incorporated into hydrogels [74] (Figure 10c). In addition, the CP/LS NPs created a dynamic redox environment to maintain the balance of catechol and quinone groups, similar to the behavior of mussels, which endowed the hydrogel with a high and repeatable adhesiveness. This conductive and adhesive hydrogel has the potential to be used for tissue regeneration and implantable bioelectronics owing to its good electrical activity and biocompatibility. In our recent study, conductive and hydrophilic dPEDOT NPs were prepared by confining the polymerization of 3,4-ethylenedioxythiophene (EDOT) with the assistance of dopamine methacrylate (DMA) oligomer templates. The dPEDOT NPs were then incorporated into a PAM hydrogel, which endowed the hydrogel with high conductivity, brain-level modulus, robust adhesiveness, and an immune-evasive ability [75]. Consequently, the hydrogel can be integrated with metallic microcircuits to form nondestructive, and conformal brain-machine interfaces, enabling long-term and accurate electro-encephalographic signal acquisition and communication with brain tissue (Figure 10d). In short, the introduction of mussel inspired strategies to fabricate conductive polymer NPs can not only ensure their conductivity but also improve their integration with polymer hydrogels, which can be widely applied in biomedical applications.

## 3. Mussel-Inspired 1D Nanomaterials-Loaded Hydrogels

Unlike 0D nanomaterials, 1D nanomaterials have a high length-to-diameter ratio (e.g., nanotubes, nanowires, and nanofibers), and thus exhibit unique physicochemical properties owing to their distinct structure and size effects.

### 3.1. Carbon Nanotubes (CNTs)

**Figure 11:**
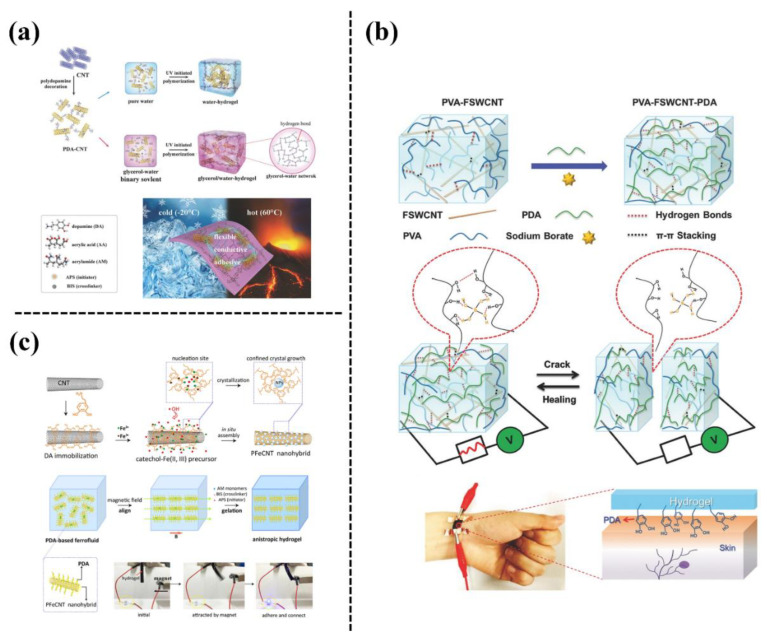
(**a**) Fabrication of a mussel-inspired conductive, adhesive, and anti-freezing/anti-heating hydrogel by co-polymerization of PDA-modified CNTs with AA and AM in a glycerol-water (GW) binary solvent [76]. (**b**) Schematic diagram of the synthesis process of a conductive, healable, and adhesive hydrogel by incorporation of a conductive PDA-functionalized SWCNTs nanotube into a dynamic supramolecular crosslinked PVA hydrogel, which can be adhered on the wrist for healthcare monitoring [77]. (**c**) Schematic of preparation of an anisotropic hydrogel based on a mussel-inspired conductive ferrofluid comprised of PDA-mediated conductive and magnetic Fe_3_O_4_-CNT nanohybrid (PFeCNT). Demonstration of the anisotropic hydrogel simultaneously possessing conductive, magnetic, and self-adhesive properties [78].

CNTs are tubular forms of carbon with diameters <1 nm and lengths ranging from a few nanometres to microns. CNTs have been used as excellent nano-reinforcing materials to endow hydrogels with improved electrical conductivity and mechanical properties. To promote the uniform distribution of CNTs in the polymer network, the surface of CNTs can be coated by catechol groups, based on a mussel-inspired functionalization strategy. Han et al. [76] designed a conductive hydrogel with extreme temperature tolerance by using PDA-coated CNTs as conducting nanofillers in a binary solvent system comprising water and glycerol (Figure 11a). Thus, the hydrogel can be used in anti-freezing or anti-heating bioelectronics and in electronic skin to collect biosignals in extreme conditions, such as skiing, polar, and desert expeditions. Liao et al. [77] also prepared a self-adhesive, self-repairing, and conductive hydrogel by incorporating PDA functionalized single-walled carbon nanotubes (SWCNTs) (Figure 11b) in a supramolecular crosslinked PVA network. The hydrogel is biocompatible, can be used as a soft strain sensor, and can be combined with a wireless transmitter to monitor human activities (bending and relaxing fingers, walking, chewing, and pulse rate). In addition, incorporating 1D nanomaterials into the polymer network can manipulate the anisotropic properties of the hydrogel. For example, Liu et al. [78] synthesized a conductive and magnetically responsive anisotropic hydrogel using CNT-iron oxide composite NPs (PFeCNT), which mimicked the directional biological tissues (Figure 11c). Based on the metal-ion chelation mechanism, ultra-small iron oxide NPs were uniformly grown on the surfaces of PDA-coated CNTs in situ to form PFeCNT. The hydrogel exhibited anisotropic mechanical and electrical properties, which had the ability to guide the migration and growth of cells under an external electrical stimulation. Thus, this type of hydrogel can be used as a cell culture platform to provide magnetic and electrical signals for regulating the targeted growth of cells and tissues in biomedical applications. In short, the catechol groups on CNTs not only facilitates their uniform dispersion in hydrogels, but also enable the in situ growth of ultra-small nanoparticles on their surface to realize the design of more complex materials, thus bringing more diverse functions.

### 3.2. Gold Nanorods (Au NRs)

**Figure 12:**
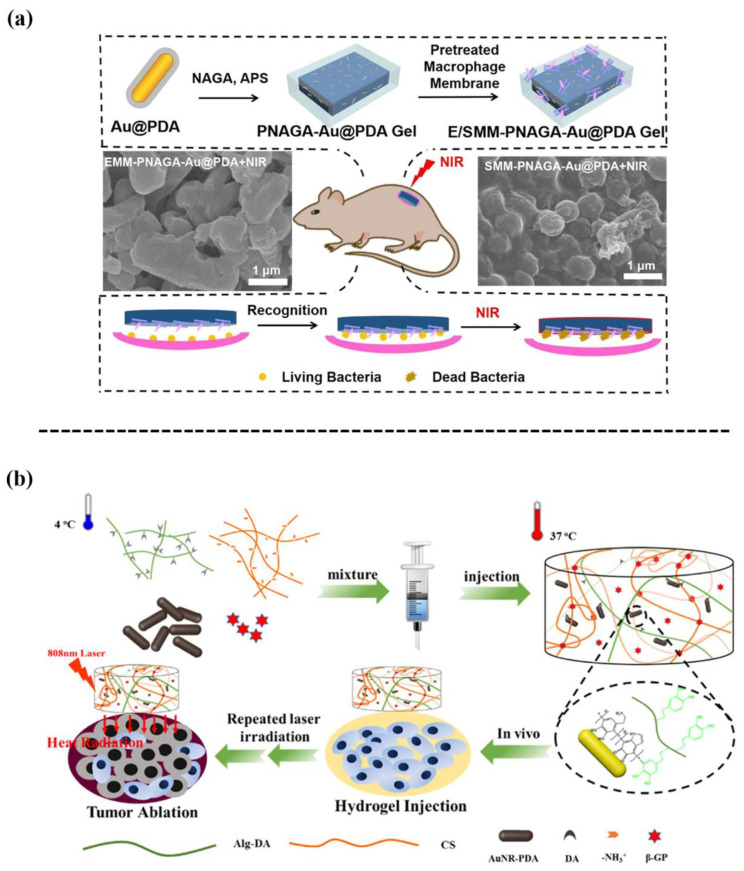
(**a**) Fabrication of nanocomposite antibacterial hydrogel with high strength and toughness by one-pot polymerization of N-acryloyl glycinamide (NAGA) mixed with polydopamine-coated gold nanorods (Au@PDA NRs) [79]. (**b**) Preparation of thermosensitive CGP/Alg-DA/AuNR hydrogels with photothermal activity for tumor therapy [80].

Compared with spherical gold nanoparticles, gold nanorods (Au NRs) show a lower energy surface plasma band, which is beneficial for photothermal antibacterial and antitumor therapies [106]. For example, Li et al. [79] prepared an antibacterial nanocomposite hydrogel (PNAGA-Au@PDA) by polymerizing a monomeric solution of N-acrylamide (NAGA) with PDA-coated gold nanorods (Au@PDA NRs). In a rat model of total skin defect repair, the PNAGA-Au@PDA hydrogel showed the synergistic effects of targeted binding to specific bacteria and photothermal-induced antibacterial activity of Au@PDA NRs, accelerating wound healing without causing secondary injury to the wound during peeling due to its excellent toughness (Figure 12a). To prevent the removal of Au NRs from local pathology sites, Zeng et al. mixed PDA-Au NRs with a thermo-sensitive injectable hydrogel (CGP/Alg-DA/AuNR hydrogels) composed of beta-glycerophosphate-bound chitosan (CGP) and dopamine-modified-alginate (Alg-DA) [80] (Figure 11b). The PDA-Au NRs can be fixed at the tumor site by the hydrogel, thus enhancing the efficacy of photothermal therapy to significantly inhibit the growth of tumor cells under multiple photothermal treatments.

### 3.3. Cellulose Nanofibers (CNFs)

**Figure 13:**
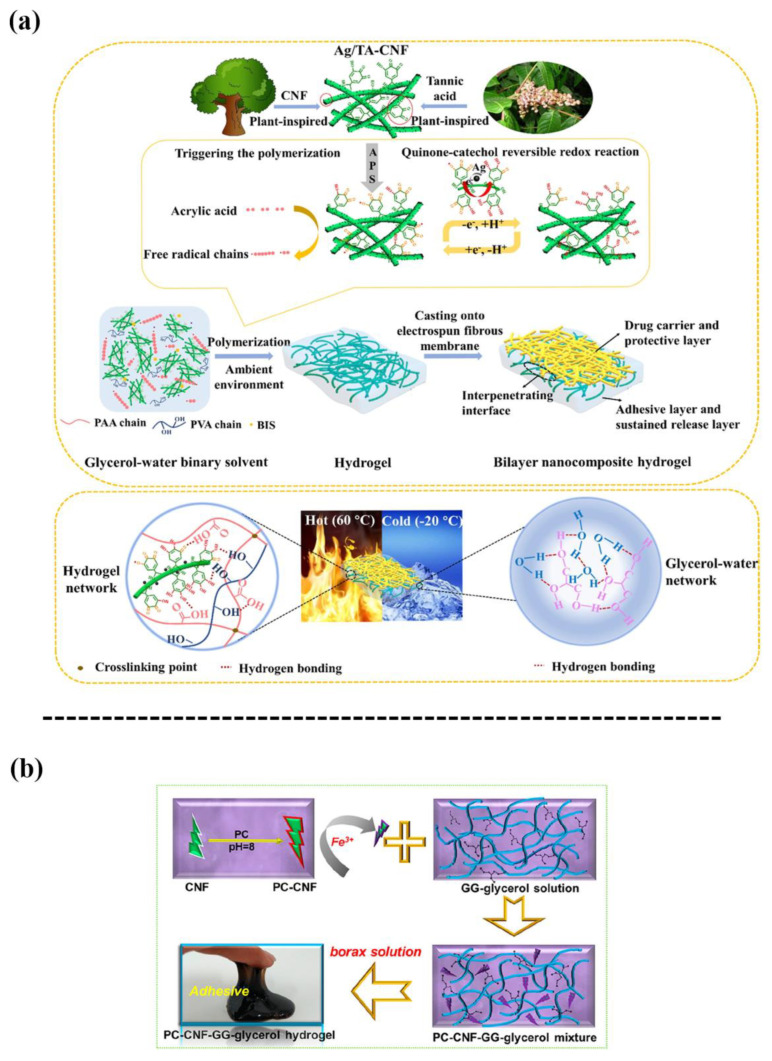
(**a**) Design strategy of a multifunctional hydrogel by co-polymerizing acrylic acid (AA), polyvinyl alcohol (PVA), and silver /TA-CNF in a glycerol-water binary solvent [81]. (**b**) Preparation of a PC-CNF-GG-glycerol hydrogel [82].

Cellulose nanofibers (CNFs) are biodegradable, green, and eco-friendly nanoscale building blocks for constructing high-performance nanocomposite hydrogels. For example, Chen et al. [81] successfully prepared hydrogels with antibacterial, antioxidant, and high-temperature resistance by using Ag NPs/TA-loaded-CNFs (Ag/TA-CNFs) to trigger free-radical polymerization of PAA and PVA (Figure 13a). The Ag/TA-CNFs in the hydrogel network facilitated dynamic reactions between the catechol and quinone groups, resulting in the repeatable adhesion property of the hydrogel. Pan et al. [82] also synthesized a guar gum (GG)-based hydrogel, which contained proanthocyanins (PC)-coated CNFs in a glycerin-water system (Figure 13b). The PC was rich in polyphenol groups, which endowed the hydrogel with excellent adhesive properties and an efficient UV-shielding ability. Thus, the hydrogel can be used in non-invasive electrodes, strain sensors, and dressings. In short, the CNFs are usually self-assembled into dense layers. The mussel-inspired strategy may be a simple, controllable, and effective method to prepare 3D composite scaffolds by uniformly dispersing the CNFs nanofibers in the hydrogel’s 3D network.

## 4. Mussel-Inspired 2D Nanomaterials-Loaded Hydrogels

The 2D nanomaterials have garnered increasing research interest since 2004 because of the availability of mechanically stripped graphene. The 2D nanomaterials have sheet-like shapes, with high surface-to-volume ratios (two dimensions > 100 nm and an ultra-thin layer), which gives them astounding electronic properties, ultrahigh specific surface areas, and excellent mechanical properties [107]. Typical examples of 2D nanomaterials include silicate nanosheets, graphene/graphene-derived nanosheets, layered double hydroxides, and phosphate-based nanosheets, which are generally employed to prepare 2D nanomaterials-loaded hydrogels with a variety of interesting properties.

### 4.1. Silicate Nanosheets

**Figure 14:**
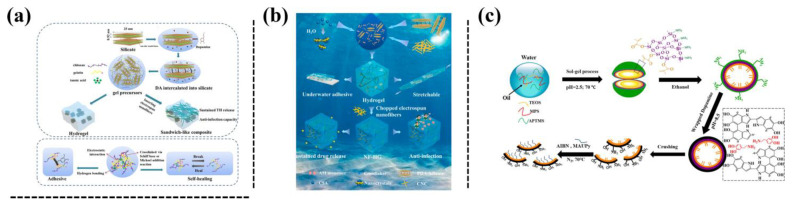
(**a**) The fabrication of multifunctional sandwich-like NF-HG based on polydopamine-intercalated silicate nanoflakes (PDA-Silicate) [83]. (**b**) The formation of an underwater adhesive and stretchable hydrogel by incorporation of PDA-Silicate nanosheets [84]. (**c**) Fabrication of the SiO_2_@PDA/PMAUPy Janus nanosheets by using an emulsion interfacial self-assembled sol-gel process [85].

Layered silicates (also called layered alkali silicates or layered polysilicates) with skeletons comprised of SiO_4_, exhibit rich intercalation chemical reactions because of the presence of silanol groups. For example, Chen et al. [83,84] designed a series of adhesive hydrogels using a mussel-inspired adhesive mechanism. In their studies, silicate nanosheets induced the oxidization of dopamine to form PDA-intercalated silicate (PDA-silicate), and then the PDA-silicate was incorporated into the hydrogel, which led to outstanding adhesiveness (Figure 14a,b). Li et al. [85] synthesized Janus silica nanosheets (SiO_2_@PDA/PMAUPy JNs) by grafting 2-(3-(6-Methyl-4-oxo-1,2,3,4-tetrahydropyrimidin-2-yl)ureido)ethyl methacrylate (MAUPy) onto the PDA-modified silicon spheres (SiO_2_ JHs) using the Pickering emulsion method, and then the prepared Janus silica nanosheets were further applied to prepare nanocomposite PAA hydrogels (Figure 14c). The resulting hydrogels showed self-healing (healing ratio of 92.6%) and impressive mechanical properties (strain of about 411.0%, stress of about 4.1 MPa), showing their potential applications as smart flexible sensors.

### 4.2. Graphene Oxide Nanosheets

**Figure 15:**
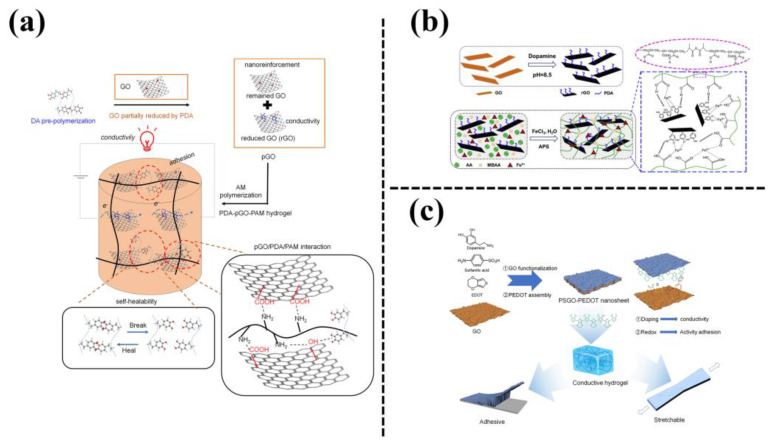
(**a**) Preparation of a conductive, self-adhesive, and self-healable hydrogel by introducing PDA-partially-reduced GO (pGO) nanosheets into a PAM hydrogel [86]. (**b**) Incorporating pGO into a PAA network to form a mussel-inspired conductive nanocomposite hydrogels [87]. (**c**) Fabrication of the hydrophilic, conductive, and redox-active sandwich-like PSGO-PEDOT nanosheet and its incorporation into a PAM network to form a hydrogel with high conductivity, adhesiveness, and stretchability [88].

Graphene is a promising 2D material in the preparation of high multifunctional composites owing to its large specific surface area, high modulus, good conductivity, and biocompatibility [108]. However, graphene nanosheets tend to aggregate in aqueous solution, which causes an uneven distribution in the hydrogel network. Graphene oxide (GO) nanosheets have abundant functional groups (hydroxyl group, carboxyl group, and epoxy group), which can be evenly dispersed in an aqueous solution, and therefore GO nanosheets are widely used for the preparation of composite hydrogels with improved mechanical properties. However, GO nanosheets exhibit poor electrical conductivity compared with graphene. Thus, synthesizing hydrogels with good mechanical and desirable electrical properties at the same time is challenging. Mussel-inspired dopamine is a green reducing agent to obtain reduced graphene oxide (rGO) nanosheets with high compatibility with hydrophilic polymers. Composites of conductive nanosheets and hydrogels are promising candidates for the next generation of soft bioelectronics, which have the potential to be used in artificial intelligence, human-computer interaction, and wearable personal healthcare devices. For instance, Han et al. [86] reported a mussel-inspired strategy to partially reduce GO nanosheets to conductive pGO nanosheets, and then designed a conductive hydrogel with integrated stretchability, self-adhesiveness, and self-healablility (Figure 15a). They demonstrated the application of the hydrogel in self-adhesive motion sensors and as self-adhesive electrodes for stable signal detection. Jing et al. [87] also developed a stretchable, flexible, and highly sensitive hydrogel strain sensor by incorporating pGO into a strong and stable polyacrylic acid (PAA) network (Figure 15b). The hydrogels thus exhibited strain sensitivity owing to an electrical pathway provided by the pGO, and detected a wide range of human movements. Gan et al. [88] employed pGO nanosheets as templates for the self-assembly of PEDOT to form highly conductive and sandwich-structured PSGO nanosheets (Figure 15c). The PSGO nanosheets were rich in hydrophilic groups, which were further incorporated into a PAM network to form a PSGO-PEDOT-PAM hydrogel with excellent electrical conductivity and tissue adhesiveness. This hydrogel was successfully used as an adhesive electronic skin for detecting electrocardiogram, electroencephalogram, and electromyogram signals.

**Figure 16:**
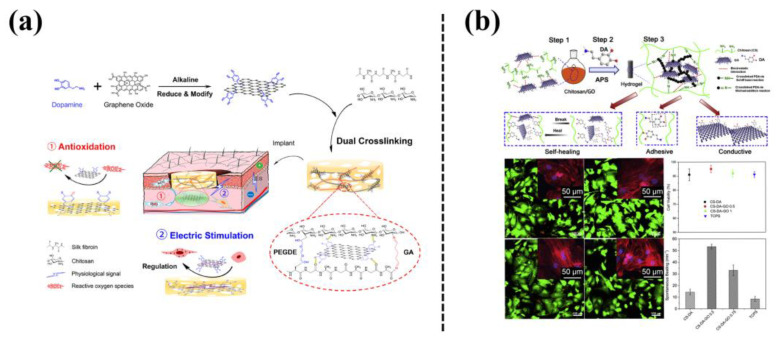
(**a**) Preparation of a dual-crosslinked pGO-CS/SF scaffold for repairing skin wound defects [109]. (**b**) Schematic of preparation of a CS-DA-GO composite hydrogel. Primary cardiomyocytes derived from human embryonic stem cells were seeded onto CS-DA-GO composite hydrogels to evaluate the biocompatibility of the hydrogels for cardiac tissue engineering [110].

In addition, the pGO nanosheets composite hydrogels have the potential to be used in tissue engineering of electroactive tissues (heart tissue, skeletal muscle, and nerves). For example, Tang et al. [109] incorporated PDA-reduced graphene oxide (pGO) into a chitosan/silk fibrin interpenetrated network to fabricate a biopolymer-based composite pGO-CS/SF hydrogel scaffold as a wound dressing (Figure 16a). The introduction of pGO nanosheets enhanced the mechanical properties of the hydrogel. Second, the uniformly distributed pGO provided an electrical pathway in the hydrogel. Third, the pGO in the hydrogel acted as antioxidants to remove reactive oxygen species (ROS). Consequently, such an electroactive and antioxidative pGO-CS/SF scaffold effectively promoted wound healing in the rat models of full-thickness skin defects. Jing et al. [110] also prepared a self-adhesive, self-healing, and conductive hydrogel by incorporating pGO into a chitosan (CS) network (Figure 16b). As a result, the electrical conductivity of the hydrogel was similar to that of the natural myocardium. Furthermore, the pGO promoted the adhesion of cardiomyocytes. The cardiomyocytes that adhered again, exhibited a high spontaneous beating rate.

### 4.3. Clay Nanosheets

**Figure 17:**
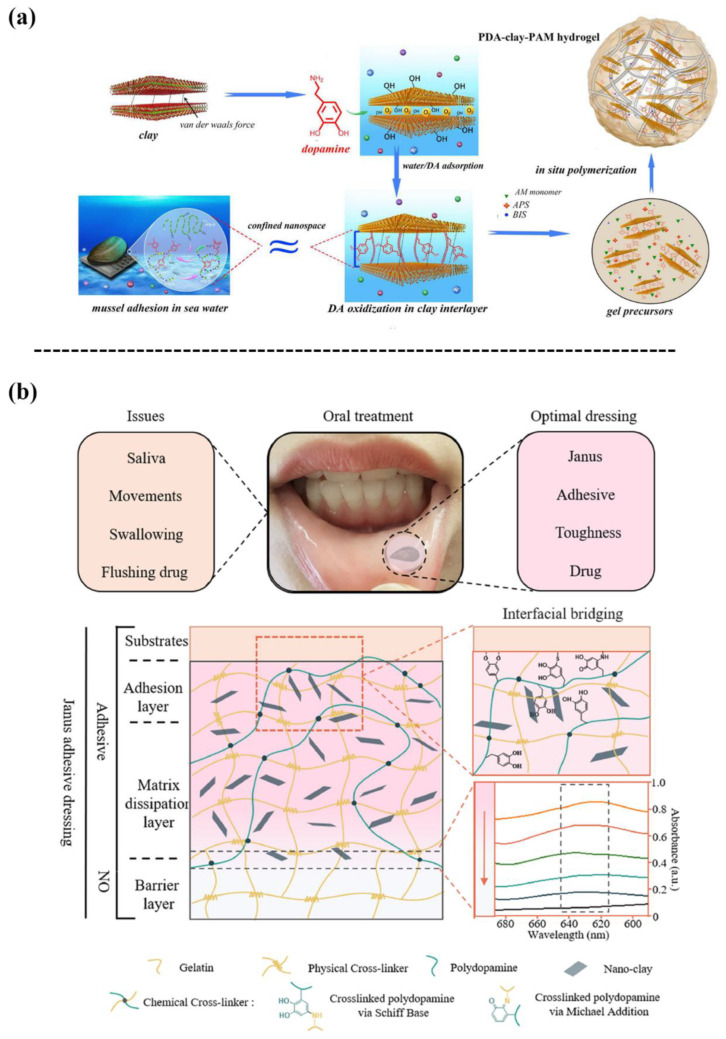
(**a**) Design strategy for the preparation of a mussel-inspired adhesive polydopamine-clay-polyacrylamide (PDA-clay-PAM) hydrogel by the confined oxidation of dopamine [89]. (**b**) Fabrication of a mussel-inspired Janus mucosal dressing with a tough and adhesive hydrogel composed of gelatin, polydopamine, and nano-clay to achieve drug release [90].

In contrast to carbon-based 2D nanomaterials, clay nanosheets are primarily composed of minerals that are present in the human body. Clay nanosheets exhibit excellent biocompatibility and biodegradability under physiological conditions. Generally, clay nanosheets have a layered structure with negative charges on each face and positive charges along the edge of the nanosheet, which give them a high drug loading ability, water stability, and enhanced interactions with biological components, such as biopolymers, proteins, biomolecules, and cells [111,112,113]. For example, Becher et al. [114] loaded cisplatin, 4-fluorouracil, and cyclophosphamide into Laponite nanocomposite hydrogels, and then evaluated the drug release behavior of the resulting nanocomposite hydrogels in a breast cancer model and an ovarian cancer model. To prevent bacterial infection from occurring on the implant surface, Wang et al. [115] prepared a multilayer film based on MMT and hyaluronic acid (HA) for on-demand release of antibiotics (gentamicin). The films exhibited high gentamicin loading capacities due to the high adsorption ability of the positively charged MMT. In our group, we fabricated a tough hydrogel with repeatable and long-lasting adhesiveness based on PDA-intercalated clay nanosheets, in which DA molecules were partially oxidized between the confined domain space of clay nanosheets, resulting in the retention of abundant catechol groups [89] (Figure 17a). The resulting hydrogel also possessed a high affinity for cell attachment and proliferation, which means it can be used as a dressing material to accelerate skin wound healing. An et al. [90] also prepared a wet-adhesive nanocomposite hydrogel comprised of gelatin, nano-clay, and dopamine, and they utilized the hydrogel as a mucosal dressing and drug delivery system (Figure 17b). The hydrogel loaded with dexamethasone effectively enhanced the healing effect of an oral ulcer, as demonstrated in a rat model of oral ulcer.

### 4.4. Talc Nanosheets

**Figure 18:**
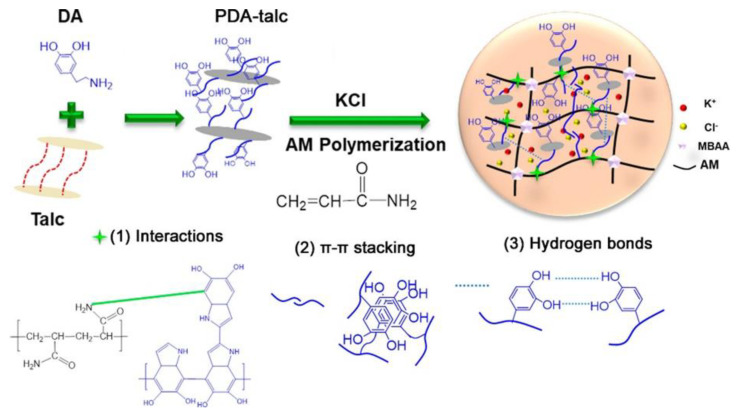
Schematic of the fabrication process of the DA-talc-PAM hydrogel [91].

Talc is a 2D layered, low-cost, environmentally friendly, and stable naturally abundant mineral [116]. Talc nanosheets easily shear and exhibit good self-lubrication properties through the weak binding of the van der Waals cambium lamellar structure. In addition, 2D talc nanosheets have high crystallinity, electrical conductivity, high thermal stability, and good adsorption properties, and so can be employed as functional nanofillers to fabricate nanocomposite hydrogels. For example, Jing et al. [91] synthesized a self-healing adhesive hydrogel by adding PDA-coated talc nanosheets to a PAM hydrogel, which is used as a strain sensor for human movement monitoring (Figure 18). Talc-induced partial oxidation of dopamine and PDA-modified talc particles were uniformly dispersed in the PAM hydrogel, which significantly enhanced the mechanical and adhesive properties of the hydrogel. When assembled as a strain sensor, the hydrogel accurately detected various human movements, such as finger, knee, elbow bending, and even deep breathing.

### 4.5. Phosphate-Based Nanosheets

**Figure 19:**
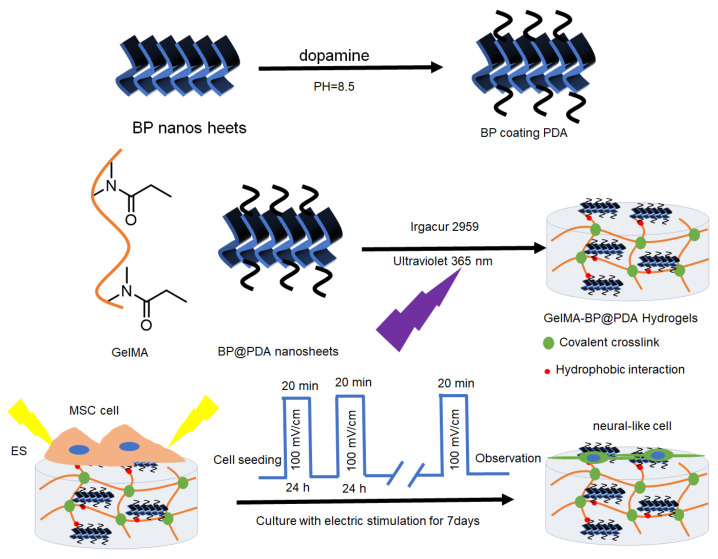
Schematics of GelMA-BP@PDA hydrogels. The surface modification of BP nanosheets by PDA. Incorporation of the PDA-BP (BP@PDA) nanosheets into a GelMA network. Schematic of regulating the growth and differentiation of MSCs by conductive GelMA-BP@PDA hydrogel under external electrical stimulation [92].

Phosphate-based 2D nanosheets, such as black phosphorus (BP), silicon phosphide, and germanium phosphide (GeP), have received significant interest in recent years owing to their excellent physical properties in electronic and energy devices. In addition, most phosphate-based nanosheets can eventually be degraded in physiological environments, and their degradation products are harmless to the human body [117,118,119]. Therefore, phosphate-based nanosheets are promising biodegradable multifunctional nanomaterials for use in therapeutic diagnostics and tissue engineering. Among them, BP has been employed in a variety of biomedical applications, including smart drug carriers for cancer therapy, photothermal reagents, photodynamic therapy reagents, reactive oxygen scavengers, and bioactive materials for bone regeneration [120,121,122]. For example, Xu et al. [92] synthesized a conductive hydrogel by incorporating PDA-modified BP nanosheets (BP@PDA) into a GelMA hydrogel for repairing electroactive tissues (Figure 19). Their results showed that the PDA on the surface of the BP nanosheets not only preserved the cell affinity but also improved the biostability of the BP nanosheets in the physiological environment. Consequently, such a BP@PDA-GelMA hydrogel exhibited excellent biocompatibility, conductivity, and biodegradability, which enhanced the differentiation of mesenchymal stem cells into the neural-like cells under electrical stimulation.

**Figure 20:**
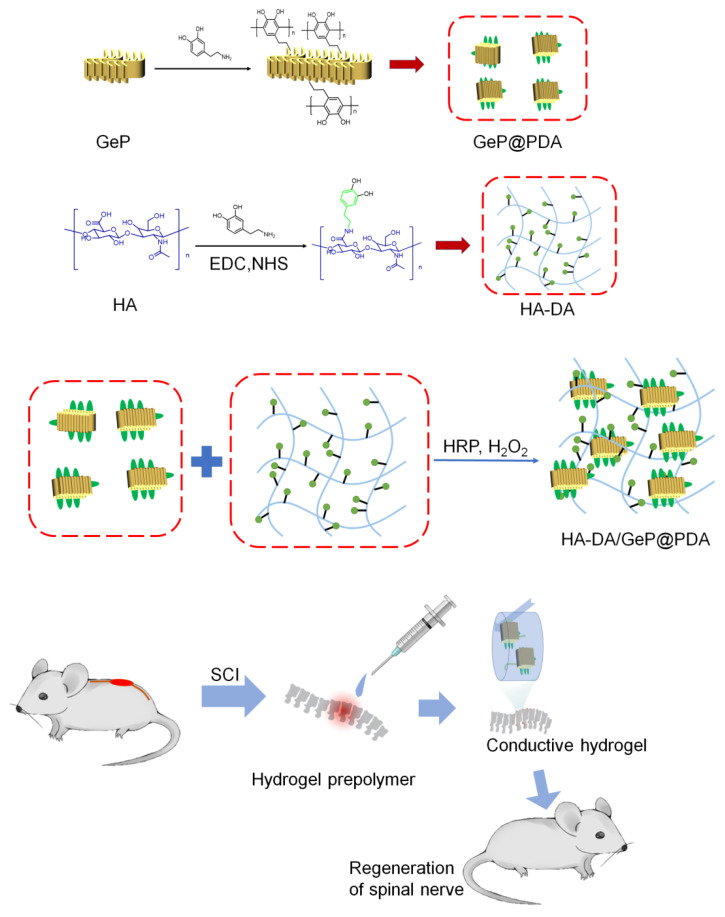
Preparation of GeP@PDA nanosheets, HA-DA polymers, and HA-DA/GeP@PDA conductive hydrogels. Application of the injectable and conductive HA-DA/GeP@PDA hydrogel for spinal cord injury repair [93].

Germanium phosphide (GeP) is another emerging 2D nanomaterial, which theoretically exhibits higher thermodynamic stability, higher carrier mobility, and a wider adjustable band gap than BP nanosheets [123]. Xu et al. [93] also employed PDA to enhance the biostability and biocompatibility of GeP nanosheets (GeP@PDA), and then prepared a biohybrid hydrogel by integration (GeP@PDA) into a horseradish peroxidase (HRP)/H_2_O_2_ cross-linked HA-DA network (Figure 20). The resulting HA-DA/GeP@PDA hydrogel was injectable, biodegradable, conductive, and adhesive, which means it could regulate immune response, and promote angiogenesis and neurogenesis to improve the recovery of motor function, as demonstrated in a rat spinal cord injury complete transection model.

### 4.6. Metal Carbides and Nitrides Nanosheets

**Figure 21:**
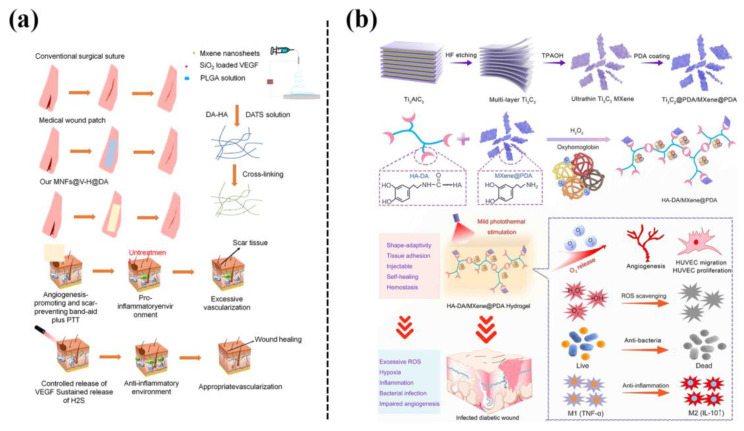
(**a**) Preparation of a MNFs@V-H@DA hydrogel, and wound healing process treated by the MNFs@V-H@DA hydrogel [94]. (**b**) Fabrication of HA-DA/MXene@PDA hydrogel and its therapeutic effects on infected diabetic wound healing, including supplying O_2_, scavenging ROS, eradicating bacteria, and inhibiting inflammation [95].

Mxene is a layered two-dimensional transition metal material, including transition metal carbide, nitride and carbonitride, expressed as M_n_ + _1_X_n_, where M is a transition metal, such as Ti or Nb, and X is carbon and/or nitrogen [124]. Mxene nanosheets have also been widely studied in the biomedical field due to their unique chemical and physical properties, such as electrical conductivity, photothermal conversion ability, and drug loading ability. For example, Ye et al. [125] exploited their electrical conductivity in a conductive heart patch (ECP) developed based on a cryogel composed of MXene Ti_2_C, dopamine-N′,N′-methylene-diacrylamide (DOPA-MBA), methacrylate-gelatin (MA-G), and polyethylene glycol diacrylate. The introduction of dopamine and MXene Ti_2_C endowed the cryogel with strong adhesion and a good retention ability to heart cells, excellent mechanical properties, and a high electrical conductivity, to match the natural heart muscle. Jin et al. [94] utilized the photothermal conversion ability of Mxene nanosheets and integrated the Mxene nanosheets into a dopamine-hyaluronic acid hydrogel (H) to develop a near-infrared photothermal responsive band-aid that can control the release of VEGF (Figure 21a). The band-aid achieved scarless wound healing in a mouse model of back wound. Li et al. [95] fabricated an injectable hydrogel based on dopamine-grafted hyaluronic acid (HA-DA) and PDA-coated Ti_3_C_2_ MXene nanosheets through the oxidative coupling of catechol groups (Figure 21b). The PDA-coated-MXene nanosheets in the hydrogel could kill bacteria through their photothermal conversion ability and scavenge ROS to relieve oxidative stress. In addition, catechol groups endowed hydrogels with anti-inflammatory properties and regulated macrophage polarization. Finally, the hydrogel accelerated the healing of infected diabetic wounds, as demonstrated by a full-thickness cutaneous injury mouse model.

In short, the common 2D nanomaterials, including silicate nanosheets, GO nanosheets, clay nanosheets, talc nanosheets, phosphate-based nanosheets, metal carbide and nitride nanosheets are summarized. These 2D nanomaterials can prevent the overoxidation of catechol groups by using their limited layered space to retain enough active groups. In addition, the addition of these 2D nanomaterials provides the hydrogels with conductivity, drug loading ability, lubrication, or photothermal conversion properties, enhancing the applicability of nanocomposite hydrogels in a variety of complex environments.

## 5. Conclusions and Outlook

Developing nanocomposite hydrogels with desired functionalities is critical for meeting the requirements of the ultimate applications, such as regenerative medicine and drug delivery, etc. This review has summarized the recent advances of nanocomposite hydrogels from the aspects of the dimensions of different nanomaterials (0, 1, and 2D nanomaterials). The review systematically discussed the incorporation of nanomaterials, including porous/solid/hollow nanoparticles, nanofibers, nanosheets, nanowires, nanotubes, and nanofibers into polymeric hydrogels based on a mussel-inspired strategy. In addition to PDA, the ability of a series of natural polyphenols with the same or similar functional groups to modify or hybridize nanomaterials for the fabrication of nanocomposite hydrogels blocks was also discussed. The catechol or polyphenol groups are primarily introduced in three ways: (1) combining with nanomaterials; (2) combining with the hydrogel matrix; (3) introducing into the nanocomposite hydrogel as an independent unit in the form of a PDA. The introduction of catechol or polyphenol groups significantly enhances the functional diversity of the nanocomposite hydrogels, such as ROS clearance, cell affinity, tissue adhesion, or the ability to enhance nanozyme activity inside the hydrogel. Although the basic properties of catechol and polyphenol groups, such as adhesive and anti-oxidative abilities, have been extensively studied, novel biological functions resulting from these properties are yet to be developed in disease-specific environments. In addition, the catechol/polyphenol-functionalization of nanomaterials offers the application of various nanomaterials that would otherwise not bind to hydrogels. The combination of catechol or polyphenol groups with nanomaterials studied thus far is relatively simple. More complex nanocomposite systems should be designed to make the functionality of catechol groups more widely discovered and explored.

## Figures and Tables

**Table 1:** Representative nanocomposite hydrogels.

Class	Nano- Materials	Modification Strategy	Properties	Behaviors	References
0DM	HA NPs	Surface coated by PDA	Nanograde solid gold particle	Osteogenic activity	[48,49,50]
	MNPs	Surface coated by PDA	Nanograde solid gold particle	Magnetothermal conversion	[51,52]
	silica NPs	Surface coated or hybridized by PDA	Nano-mesoporous structure	Drug-loading capability	[53,54,55,56,57]
	MOF	Surface coated or hybridized by PDA	Nano-Porous structure	Drug-loading capability	[58,59,60]
	Ag NPs	Reduced by PDA	Nanograde solid gold particle	Antimicrobial ability	[61,62,63,64]
	PDA-NPs	Self-oxidized	Nanograde solid gold particle	Drug-loading capability, photothermal property	[65,66,67,68,69,70,71]
	CPs NPs	Hybridized by PDA	Nanograde solid gold particle	Electrical conductivity	[72,73,74,75]
1DM	CNTs	Surface coated by PDA	Hollow fibrous structure with nano radial size	Electrical conductivity	[76,77,78]
	Au NRs	Surface coated by PDA	Linear nano-rods	Photothermal property	[79,80]
	CNFs	Surface coated by PDA	Cellulose nano-rods	Mechanical enhancement performance	[81,82]
2DM	Silicate NSs	Intercalated by PDA	Single or multiple layers	Drug-loading capability	[83,84,85]
	Graphene oxide NSs	Reduced by PDA	Single or multiple layers	Drug-loading capability, electrical conductivity	[86,87,88]
	Clay NSs	Intercalated by PDA	Single or multiple layers	Drug-loading capability	[89,90]
	Talc NSs	Surface coated by PDA	Single or multiple layers	Lubricating property	[91]
	Phosphate-based NSs	Surface coated by PDA	Single or multiple layers	Drug-loading capability, photothermal property, photodynamic property	[92,93]
	Metal carbides and nitrides NSs	Surface coated by PDA	Single or multiple layers	Drug-loading capability, photothermal property, electrical conductivity	[94,95]

## Data Availability

Data sharing is not applicable to this article as no new data were created or analyzed in this study.
